# Variability of myocardial perfusion dark rim Gibbs artifacts due to sub-pixel shifts

**DOI:** 10.1186/1532-429X-11-17

**Published:** 2009-05-27

**Authors:** Pedro Ferreira, Peter Gatehouse, Peter Kellman, Chiara Bucciarelli-Ducci, David Firmin

**Affiliations:** 1 National Heart and Lung Institute, Imperial College, London, UK; 2 Royal Brompton Hospital, London, UK; 3 National Institutes of Health, Bethesda, MD, USA

## Abstract

**Background:**

Gibbs ringing has been shown as a possible source of dark rim artifacts in myocardial perfusion studies. This type of artifact is usually described as transient, lasting a few heart beats, and localised in random segments of the myocardial wall. Dark rim artifacts are known to be unpredictably variable. This article aims to illustrate that a sub-pixel shift, i.e. a small displacement of the pixels with respect to the endocardial border, can result in different Gibbs ringing and hence different artifacts. Therefore a hypothesis for one cause of dark rim artifact variability is given based on the sub-pixel position of the endocardial border. This article also demonstrates the consequences for Gibbs artifacts when two different methods of image interpolation are applied (post-FFT interpolation, and pre-FFT zero-filling).

**Results:**

Sub-pixel shifting of *in vivo *perfusion studies was shown to change the appearance of Gibbs artifacts. This effect was visible in the original uninterpolated images, and in the post-FFT interpolated images. The same shifted data interpolated by pre-FFT zero-filling exhibited much less variability in the Gibbs artifact. The *in vivo *findings were confirmed by phantom imaging and numerical simulations.

**Conclusion:**

Unless pre-FFT zero-filling interpolation is performed, Gibbs artifacts are very dependent on the position of the subendocardial wall within the pixel. By introducing sub-pixel shifts relative to the endocardial border, some of the variability of the dark rim artifacts in different myocardial segments, in different patients and from frame to frame during first-pass perfusion due to cardiac and respiratory motion can be explained. Image interpolation by zero-filling can be used to minimize this dependency.

## Background

Myocardial perfusion imaging with magnetic resonance, combined with other Cardiovascular Magnetic Resonance scans such as Late Gadolinium Enhancement, is developing into an alternative to nuclear medicine [1-5]. There is however a well-known dark rim artifact (DRA) that complicates diagnosis and quantification. There have been several possible mechanisms described in literature that can explain DRAs including cardiac motion during image acquisition [6], Gibbs ringing in the subendocardial border due to a finite resolution [7], non-uniform k-space weighting resulting in point spread function distortion [1]. Recently main field distortion during first-pass was measured and shown likely to be insufficient to cause susceptibility artifacts in typical 1.5 T perfusion protocols [8].

In this work we are going to focus only in the contribution that Gibbs artifact makes to DRAs in perfusion studies. Cardiac motion tends to cause artifacts more toward mid-wall in the myocardium [6], and non-uniform k-space weighting point-spread function distortion [1] was assumed negligible for the perfusion setup used in this study.

The Gibbs ringing artifact (also known as truncation artifact) is present at bright borders such as the endocardial border during the first-pass and it manifests as signal intensity oscillations with distance from the border. As a rule of thumb, Gibbs ringing becomes a noticeable problem when the border is so sharp that its width is equal or smaller than the true pixel size in the direction across the border. The Gibbs oscillation nearest to the border has an amplitude of 9% of the intensity change across the border (i.e. 9% undershoot and 9% overshoot) which is independent of image resolution. Although 9% might seem relatively small, it results in an 18% variation in the subendocardium for a typical blood/myocardium signal ratio of 3/1 during LV (Left Ventricle) first-pass, and this can be very noticeable. Gibbs ringing is therefore important in generating DRAs, and it is exactly during early contrast enhancement (i.e. while the LV blood is still much brighter than the myocardium) that mild perfusion defects are diagnosed. DRAs are commonly described as transient lasting a few heart beats [9,10]. Some of this transient behaviour can be explained by the DRA becoming visible only when the LV is much brighter than the myocardium, i.e. the DRA is a ringing artifact into the myocardium from the sharp edge. Therefore DRAs are of great importance because they can obscure underlying short-lived mild perfusion defects in the subendocardium and it is therefore clinically important to fully understand their properties.

**Figure 1:**
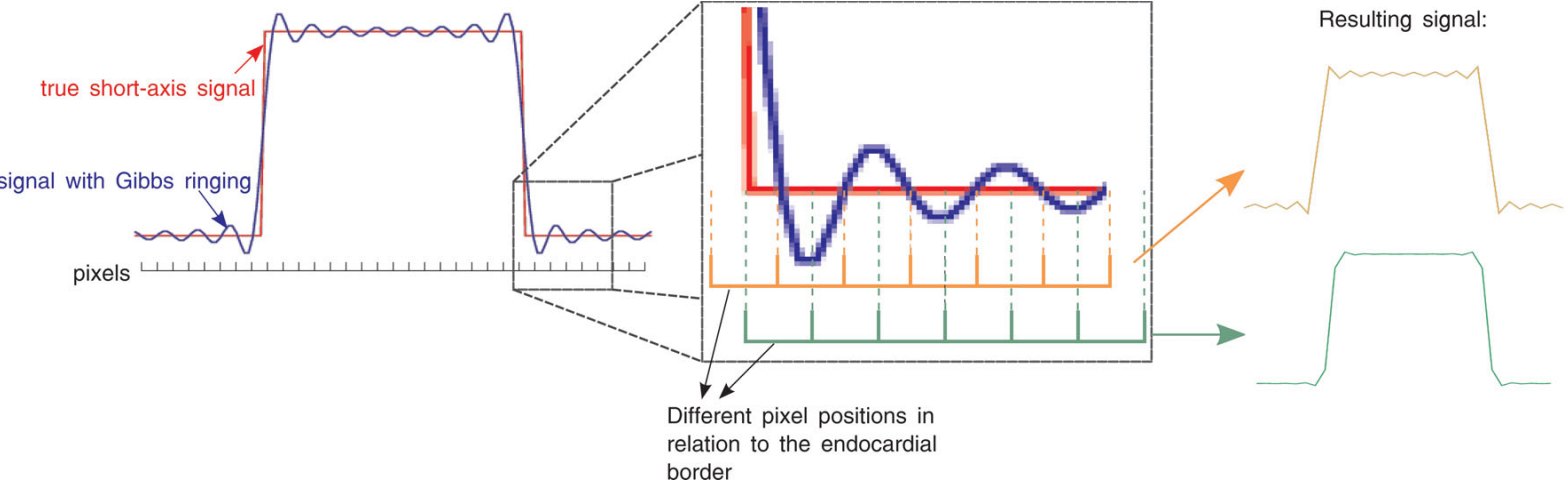
**Pixel position and Gibbs ringing**. A simulated short axis signal profile across the myocardium and LV (red). The sharp edge in the profile represents the signal contrast between the myocardium and the LV. The signal around these edges is going to be corrupted with Gibbs artifacts (blue line). Different pixel positions would measure the oscillating signal differently, since each pixel has a finite size, integrating the signal inside its corresponding position. Orange and green represent different pixel positions and the respective signal profile measured. Gibbs ringing is much more visible in the orange pixel position that on the green pixel position. This figure does not consider any image interpolation effects.

This article illustrates a source of DRA variability based on the sub-pixel position of the endocardial border. The exact position of the endocardial border with respect to a pixel will determine the amount of Gibbs artifacts visible near that border, i.e. by introducing a relative shift of the pixels by a distance smaller than the pixel size (sub-pixel shifts) the amount of visible Gibbs ringing can be affected. This is illustrated in Figure 1, on the left it is shown a simplified signal profile that could be found across a short axis of the heart, crossing the centre of the LV blood pool and the myocardial wall on both sides (red line). The sharp step of the line profile corresponds to the endocardial border on each side. Because of the limited spatial resolution of a typical perfusion image acquisition, the signal around these edges would actually be corrupted with Gibbs artifacts (blue line). Figure 1 shows how different pixel positions would result in the oscillating signal being displayed very differently. This occurs because each pixel displays the sum of the signal within its finite size (orange and green represent different pixel positions and the respective pixel values obtained). Gibbs ringing is much more prominent for the orange pixel position than for the green pixel position. (This illustration is over-simplified neglecting any point-spread function to make the fundamental point).

The appearance of the magnitude image is commonly improved by increasing the matrix size. This can be achieved by zero-filling the raw-data or by interpolating in the image space. Zero-filling (pre FFT (Fast Fourier Transform) interpolation) is widely used, and it has been reported to reduce partial volume artifacts and increase the resolution in the diagonal direction [11,12]. Image display programs also usually interpolate data in the image space (post FFT interpolation). This article will also demonstrate the effects on the Gibbs artifacts caused by interpolating the original image; mainly the difference between pre and post FFT interpolation.

The objective of this article is to illustrate the effect on Gibbs artifacts of introducing sub-pixel shifts in the context of myocardial perfusion imaging. To the best of our knowledge this has not been published before in the context of myocardial perfusion.

## Methods

### Phantom imaging

For a practical example of the effect which will be examined in the context of perfusion imaging, we made the following initial demonstration. Images were acquired with a 8 mm slice perpendicular to a flat boundary between a solution of Gd-DTPA and undiluted gelatine with a signal ratio of approximately 3/1 (which is similar to typical perfusion first-pass images when normal myocardium begins to enhance after the LV bolus peak). The sequence used was a balanced Steady State Free Precession: TR (time of repetition)/TE (echo time) of 1.6/1 ms; base resolution 128 × 128 pixels; pixel size 3 × 3 mm; flip angle 70°; bandwidth 890 Hz pixel^-1^. The image plane was slightly rotated with respect to the phantom's edge so that the edge was slightly misaligned in relation to the pixel orientation. This aimed to test the effect of varying border position in relation to pixels in the vertical direction, as a function of position across the image. The uninterpolated image was compared with the two approaches to interpolation described in the Introduction: 1) the phantom image was interpolated in MATLAB (Mathworks, Natick, USA-MA) using a bicubic interpolation in the image space by a factor of two in each direction; 2) the same raw data of the image was zero-filled by a factor of two in each direction before FFT.

### Simulated data

A short-axis image of the heart with an epicardial diameter of 65 mm and an endocardial diameter of 35 mm was simulated numerically using MATLAB. The LV/myocardium signal ratio was 3/1. The magnitude image was reconstructed from a finite 128 × 128 raw-data matrix, as would be obtained in practical MR imaging, and therefore is inherently corrupted with Gibbs artifacts. Sub pixel shifts were introduced by applying a linear phase slope across the simulated raw-data, which corresponds to a translation of the object being imaged. Reconstruction was repeated with appropriate phase slopes to introduce shifts of half a pixel horizontally and then vertically, in order to simulate the effects of a small sub-pixel shift in the subendocardium. A magnitude image with a lower image contrast with a ratio of 4/3 between the LV and the myocardium was also simulated, representing the normal heart after the bolus transit. No filtering was applied during any reconstruction.

All of the uninterpolated simulated magnitude images described above were also compared against two methods of interpolation, in a similar fashion to the phantom data: 1) image-based interpolation by a factor of 2 along each direction in the image space using bicubic interpolation (MATLAB), and 2) zero-filling of the raw-data by a factor of 2 along each direction before FFT.

### Patient images

By retrospective processing of patient data, sub-pixel shifts were also introduced into the raw-data of three patients who had clinical stress perfusion studies, where a DRA was visible. All the *in vivo *raw-data used was anonymised; totally anonymised data does not come under the jurisdiction of the UK Data Protection Act, and therefore consent for the anonymous use of patient data is not legally required in the UK. The perfusion sequence was a hybrid echo planar imaging (h-EPI) sequence with an EPI factor of 4; TR (time of repetition)/TE (time of first echo) of 5.1/1.02 ms; base resolution 128 pixels; pixel size 2.8 × 2.8 mm; slice thickness 8 mm; flip angle 30°; bandwidth 1860 Hz pixel^-1^; TI (time of inversion) of 90 ms using a non-selective BIR-4 saturation pulse, TSENSE with an acceleration factor of 2. Perfusion was imaged for three slices (order: basal, mid, apical) each heartbeat, at the first pass of Gd-DTPA at a dose of 0.1 mmol/Kg of body weight at an injection rate of 3 mL/s (1 M contrast agent). Images were acquired in the short-axis plane for fifty R-R intervals, with the patient holding their breath for as long as possible. Maximal hyperaemia was induced by a continuous intravenous infusion of adenosine at a rate of 140 μg/kg/min and stress perfusion images acquired after 4 minutes of adenosine infusion.

The *in-vivo *sub-pixel shifts applied had a step of 1/8^th ^of a pixel ranging from 0.125 to 0.875 of a pixel length. The unprocessed raw-data was transferred and an appropriate phase slope introduced using MATLAB before reconstructing the data back on the scanner using the same image reconstruction settings as the original images. The scanner's image reconstruction was also repeated with a zero-filling interpolation by a factor of two. The final non-interpolated magnitude images were also interpolated in the image space using MATLAB with a bicubic interpolation by a factor of two. The original and shifted magnitude images were compared visually.

The myocardial segments pointed out as DRAs were not in territories where a real perfusion defect was reported clinically. Specifically all the DRAs considered were transient in nature lasting only a few heart beats during first-pass and correlated with the LV bright blood signal.

The Gibbs artifact signal loss was measured as a percentage of the average myocardial signal in all experiments. These values were measured with a 3 pixel wide line profile perpendicular to the endocardial border in the myocardial segment being considered.

## Results

**Figure 2:**
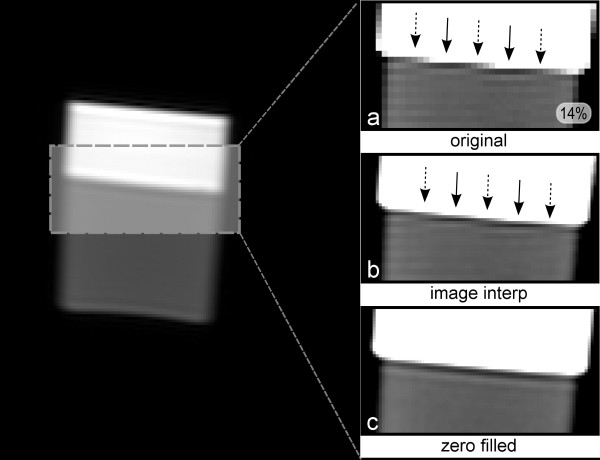
**Phantom images**. A phantom with a near horizontal flat edge between undiluted gelatine and a solution of Gd-DTPA, corresponding to a signal ratio of 1:3 in the edge boundary. a) Phantom image uninterpolated. b) Image *a *interpolated in the image space with a bicubic interpolation. The solid arrows point into the regions where Gibbs artifacts are visible, while the dashed arrows point into the in-between regions with no artifact. c) Image *a *but with zero-filling pre FFT interpolation. Figure 2 *a *(bottom right) shows the measured peak artifact signal loss in the bottom right corner.

Figure 2a–c shows the phantom edge results: uninterpolated image, the image interpolated in the image space, and the zero-filled interpolated image respectively. It can be seen, in image *a*, and *b *that the near-horizontal edge generates Gibbs artifacts at some locations along the horizontal direction (solid arrows) but not at others (dashed arrows). The artifact is seen in the single-pixel layer immediately adjacent to the bright region (simulated LV blood), to which layer all of the following descriptions will refer. The locations indicated by the solid arrows correspond to the situation shown in orange in Figure 1, while the locations of the dashed arrows correspond to the green case also shown in Figure 1. The maximum measured signal loss on the uninterpolated image (Fig 2a) was 14%. On Figure 2c, following zero filling interpolation, the artifact's variability with the edge position is less visible and this would be expected to be further reduced if more zero filling was used.

**Figure 3:**
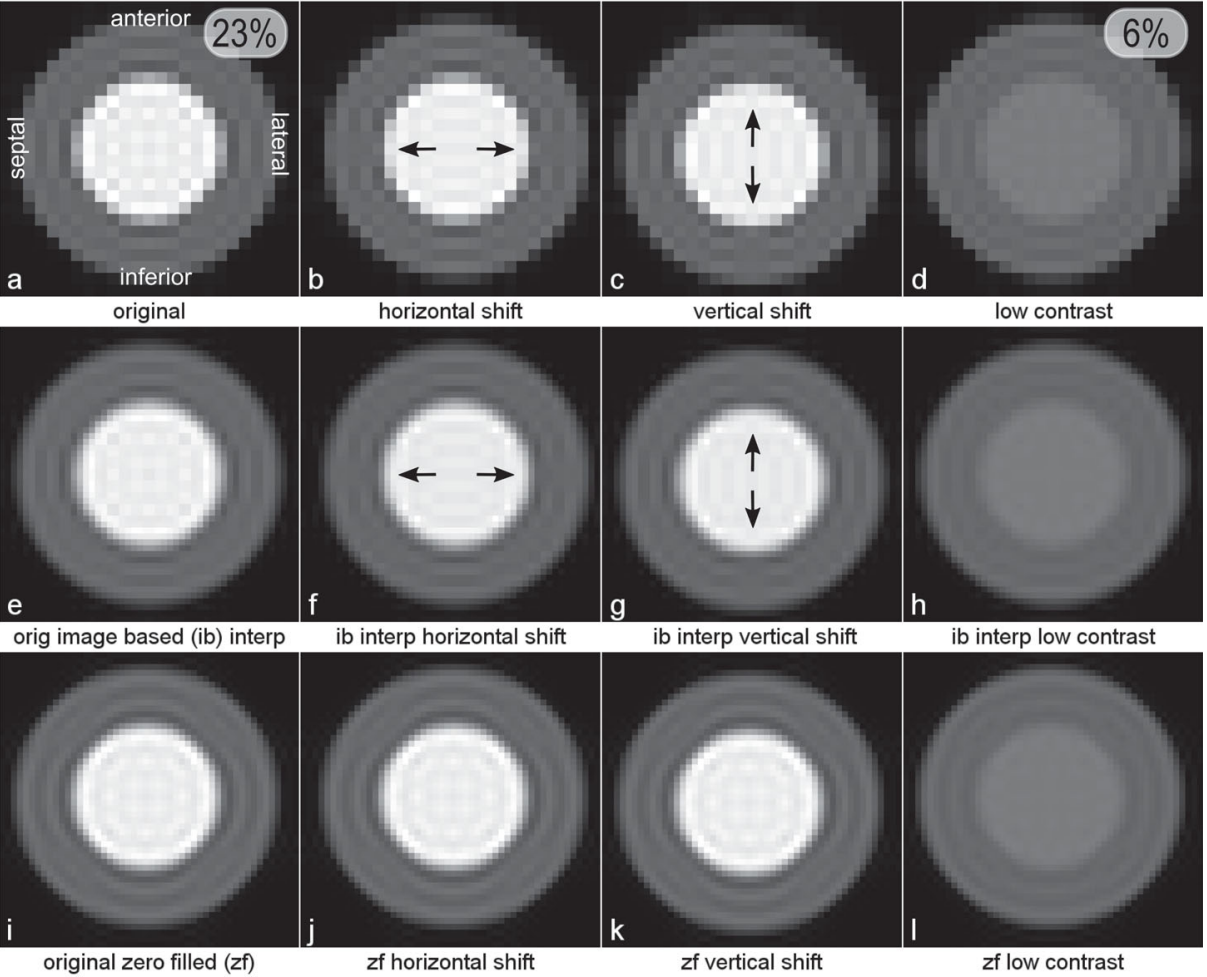
**Numerical simulation**. Top: a) numerically simulated short axis image. b) Image *a *shifted horizontally half a pixel by phase-shifting the raw-data. c) Image *a *shifted vertically half a pixel using the same method. d) Image *a *with lower LV/myocardium signal ratio. Images *e-h *are images *a-d *interpolated in the image space. Images *i-l *are images *a-d *zero-filled pre-FFT. Both figure 3 *a*, and *d *also show, in the top right corner, the correspondent measured peak artifact signal loss.

The simulated images are displayed in Figure 3. Image *a *shows Gibbs artifacts in the subendocardial layer in the anterior, septal, inferior and lateral regions. In Figure 3b, the heart has been given a half-pixel shift in the horizontal direction in relation to the original position given in *a*, and this results in a reduction of the Gibbs artifacts for the septal, and lateral segments (arrows), i.e. the regions with vertical edges. In Figure 3c the heart has been given a half-pixel shift in the vertical direction with a reduction of the Gibbs artifacts for the inferior and anterior segments (arrows), i.e. the regions with horizontal edges. However, note that at some nearby positions around the circumference, towards the diagonal regions of the subendocardium in image *b-c*, there are sometimes increases of the Gibbs artifacts with the shift; these positions can be seen to have low-Gibbs offset before the shift. Figure 3d is the same as Figure 3a except for a much lower contrast ratio between the LV blood pool and the myocardium, and this shows less prominent Gibbs artifact as expected. The measured signal losses were 23% and 6% for 3a and 3d respectively.

Figure 3e–h show the image based interpolated magnitude images of *a*-*d *respectively. This interpolation exhibits exactly the same shifting effects as in Figures 3b and 3c, i.e. a similar reduction of the artifacts in the regions pointed out by the arrows, but increased artifact at other locations.

Figure 3i–l show the zero-filled images corresponding to images *a*-*d *respectively. In this subset of images, the artifacts appear consistently regardless of the small inplane shifts of the heart.

**Figure 4:**
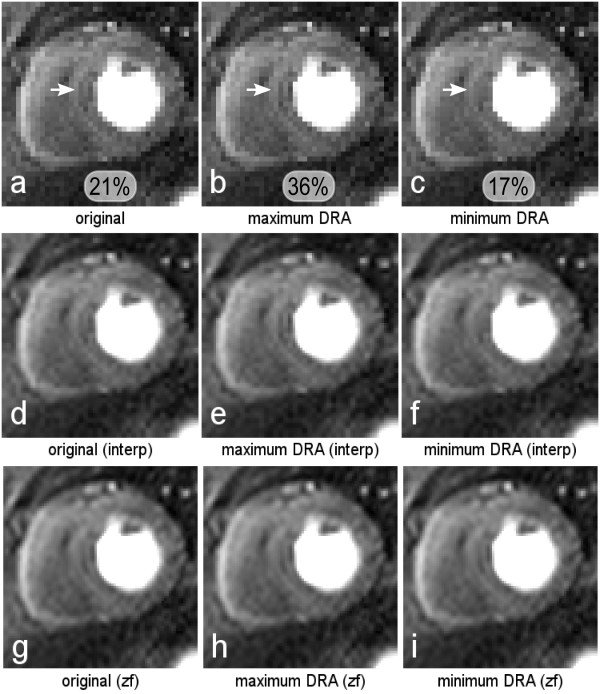
***In-vivo *I**. a) The original image retrieved from the perfusion scan, with a mild artifact in the septal region as pointed by the white arrow. b) The same magnitude image but with a shift of 0.375 pixel length in the horizontal direction, corresponding to the shift with the most prominent artifact in the same region. c) A shift of half a pixel compared to image *b *in the horizontal direction, showing a reduction of the DRA. Images *d-f*, and *g-i *show images *a-c *interpolated in the image space, and zero-filled pre-FFT respectively. Figure 4 *a-c *in the bottom, show the measured peak artifact signal loss in the region pointed by the arrows.

**Figure 5:**
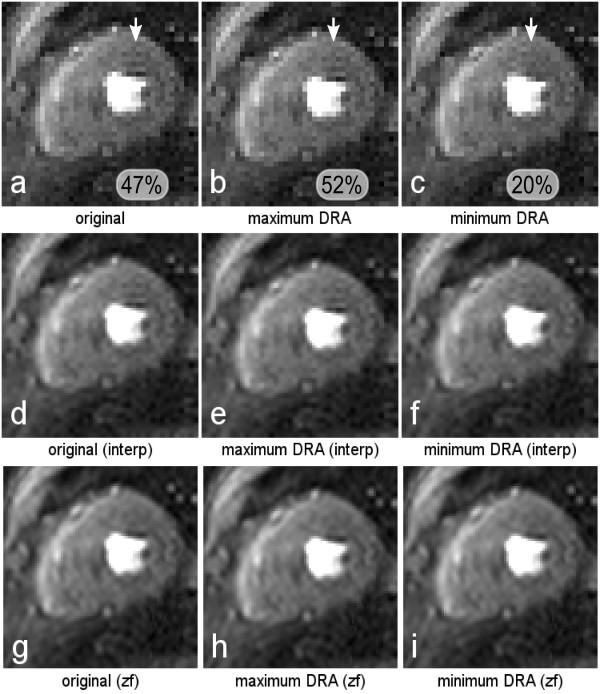
***In-vivo *II**. a) Original perfusion image with a DRA located in the anterior region of the subendocardium. b) Image *a *shifted 0.875 pixel length in the vertical direction. c) Image *a *shifted 0.375 pixel length in the vertical direction (half-pixel difference from *b*). Image *b *corresponds to the shift with the most prominent artifact, and image *c *the shift with the biggest DRA reduction. Images *d-f*, and *g-i *show the post-FFT and pre-FFT interpolations of images *a-c*. Figure 5 *a-c *in the bottom, also show the measured peak artifact signal loss in the region pointed by the arrows.

**Figure 6:**
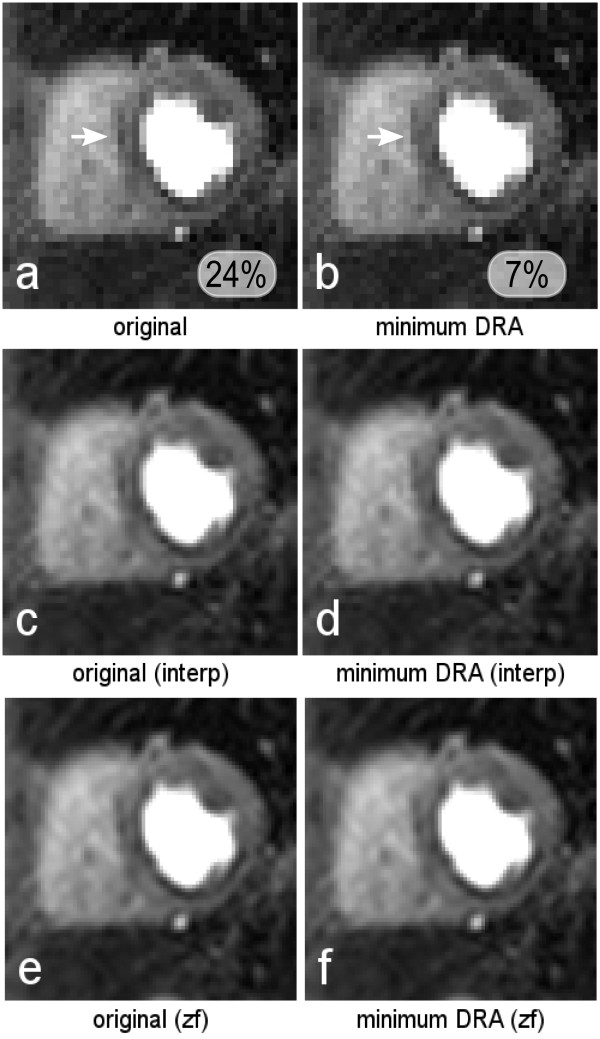
***In-vivo *III**. a) The original perfusion frame with an artifact in the septal region, and is an example where the original boundary was exactly at a location with the most prominent DRA. b) corresponds to image *a *shifted in the horizontal direction by half a pixel length, showing a reduction of the artifact. Figures *c-d *and *e-f *are the respective post-FFT and pre-FFT interpolations of figures *a-b*. Figure 6 *a*, and *b *in the bottom, show the measured peak artifact signal loss in the regions pointed by the arrows.

Results from the *in-vivo *sub-pixel shifts are shown in Figures 4, 5 and 6. Figure 4a shows the original uninterpolated image retrieved from the perfusion scan for one patient, with an artifact in the septal region as pointed out by the white arrow. After testing several sub-pixel shifts, Figure 4b shows the same magnitude image but with a shift of 0.375 pixel length in the horizontal direction, corresponding to the shift with the most prominent artifact in the same region. Figure 4c shows a shift of half a pixel compared to image *b *in the horizontal direction, showing a reduction of the DRA. The measured signal loss in the DRA was 21%, 36% and 17% for *a*, *b *and *c *respectively. Figure 4d–f, and 4g–i show images *a-c *interpolated in the image space, and zero-filled pre-FFT respectively; the effects noted in Figures 4b and 4c are replicated in Figures 4e and 4f with image-based interpolation, but the variability of Gibbs artifact is highly reduced by zero-filling interpolation (Figures 4g–i).

Figure 5a shows another *in-vivo *example where the DRA is located in the anterior region of the subendocardium. The shifts (in the vertical direction) shown in Figure 5b and 5c are 0.875 and 0.375 pixel length respectively. Figure 5b corresponds to the shift with the most prominent artifact, and figure 5c the shift with the biggest DRA reduction. The measured signal loss in the DRA was 47%, 52% and 20% for *a*, *b *and *c *respectively. Figures 5d–f, and 5g–i show the post-FFT and pre-FFT interpolations respectively. Post-FFT interpolation again replicates the uninterpolated case whereas pre-FFT interpolation greatly reduces the variability.

Figure 6a shows a third example of a perfusion scan, this time with an artifact in the septal segment, and is an example where the original mid-septal endocardial boundary happened to be in the position with the most prominent DRAs. Figure 6b corresponds to image *a *shifted in the horizontal direction by 0.5 of a pixel length, showing a reduction of the artifact. The measured signal reduction was 24% and 7% for *a *and *b *respectively. Figures 6c–d and 6e–f are the respective post-FFT and pre-FFT interpolations of figures 6a–b. Post-FFT interpolation shows the same variability of DRA appearance as the uninterpolated images, while pre-FFT interpolation highly reduces the variability. Note that there is a real perfusion defect in the inferior segment of the myocardium, but we are interested in the mid-septal segment as marked in Figures 6a and 6b.

See also additional file 1: Animation1 where four consecutive frames during first-pass of the contrast agent bolus are shown alternating between the worst and the best shift for two artifacts located in the anterior and inferior myocardial segments.

All the *in-vivo *images shown interpolated in image space exhibit similar Gibbs artifact dependency on the edge position as shown by the non-interpolated images. This dependency is highly reduced for the images interpolated by zero-filling pre-FFT, i.e. the DRAs seem identical before and after shifting the raw-data.

## Discussion

It should be noted that this work focussed on the Gibbs artifact's contribution to perfusion DRAs and other factors that contribute to DRAs are not considered here.

This work illustrates that how the exact position of the endocardial border with respect to the image pixels can explain some of the variability of the DRA in different myocardial segments, in different patients and from frame to frame during first-pass perfusion and how this could be affected by cardiac and respiratory motion. It should be understood that the relevant sub-pixel shift is the sub-pixel remainder of any larger in-plane shift, i.e. if the heart moves by a length of three and a half pixels then the sub-pixel shift effects described here are the same as moving by half a pixel. Although it may be possible to minimise the Gibbs ringing contribution to a DRA in a particular myocardial segment by introducing a phase slope to the raw-data and repeating its reconstruction, it may also make Gibbs ringing contribute more to DRAs in other subendocardial segments (i.e. where the subpixel remainder of border location in relation to pixels differs). Also although it may appear that there is perhaps the option to identify DRAs due to Gibbs by repeating shifted reconstructions while checking consistent appearance of DRA, this idea would probably be confounded by partial-volume effects on true thin defects. For these reasons, although it might seem possible to devise a tool to evaluate the contribution of Gibbs ringing artifacts by applying shifted image reconstructions, we doubt whether this would be worthwhile.

The nature of the Gibbs artifact makes it very sensitive to sub-pixel shifts, since each oscillation lobe covers the size of one pixel. Gibbs artifacts scale with the pixel size, therefore the spatial frequency of the oscillations increases exactly with the resolution. Consequently, the sensitivity to sub-pixel shift applies at any resolution, although at higher resolution the relative sharpness of endocardial borders is likely to be reduced and this reduces the artifact. This is in line with the work done Plein et al., where a high resolution k-t-SENSE perfusion study was compared with a standard SENSE perfusion protocol, scoring less dark-rim artifacts [13].

Reducing the Gibbs artifact can be achieved by filtering the raw data, reducing the ringing drastically at the price of reducing the resolution as well, which is a high cost when considering the typical low resolutions obtained clinically by most perfusion studies.

In theory each frame of each slice in a perfusion study will image the heart in the same part of the cardiac cycle, nevertheless small differences in the position of the heart walls from frame to frame will be very likely, changing the position of the edge inside an approximate 2.5 × 2.5 mm pixel. Sub-pixel shifts are therefore a contributor to the variability of the Gibbs artifacts, in addition to the variable sharpness of the endocardial border between the LV blood pool and the myocardium from frame to frame and also between patients.

The appearance of the Gibbs artifact is not only dependent on the LV blood pool/myocardium signal ratio but also on the myocardial signal-to-noise ratio, i.e. sequences less affected by random noise, such as b-SSFP, make Gibbs artifacts easier to be seen. It is also recognized that the most sensitive time for DRA is shortly after the LV peak, when contrast agent begins to perfuse normal myocardium but the LV blood is sometimes still extremely bright. Also, a higher gadolinium concentration or the use of a 3 T field with its associated increase in SNR and CNR would therefore increase the overall visibility of Gibbs artifacts. Although a rest scan is sometimes used to determine DRAs [14-16], it is often associated with a less compact bolus due to the lower cardiac output, resulting in a smaller LV/myocardium ratio, which in addition to the effect of possible sub pixel positional differences, makes the rest scan potentially unreliable in determining Gibbs-related DRAs.

This work showed that the DRA variability is reduced by zero-filling the raw-data before FFT and reconstructing a larger matrix image, since this technique upsamples (i.e. places more interpolated pixels on each cycle of) the Gibbs signal oscillations and thereby reduces the variability of Gibbs appearance; a factor higher than 2 in the interpolation would make the zero-filled data even less dependent on the edge position [12,17]. On the other hand, if interpolation in the image space is used, the sub-pixel shift variability is the same as the original data. It is important to point out that zero-filling does not reduce the artifacts in any way; it only reduces their variability by reducing their dependency on the endocardial border position. Nevertheless a less variable artifact may be preferable, especially if quantification methods are used.

A phenomenon of Gibbs ringing interference between two close edges might also be possible, such as in the septal segment of the myocardium in-between the right and left ventricle, where the ringing from both edges interfere either destructively or constructively, decreasing or increasing the Gibbs artifacts respectively.

It was difficult to measure the *in vivo *average signal intensities accurately due to the low resolution and low SNR of the perfusion frames. Nevertheless the average of the *in-vivo *signal loss caused at the artifact regions in the images with the prominent artifact was 31% of the respective average myocardial signal.

Motion artifacts are more complicated than Gibbs artifacts, in that their signal oscillation wavelength increases with distance from the sharp edge. Considering a simplification of a constant motion and a normal Cartesian k-space filling, the signal oscillations will have a first lobe width of approximately 2 pixels for a typical perfusion sequence protocol [6]. Motion artifacts alone are therefore wider and probably less prone to be affected by sub-pixel shifts, but motion artifacts may occur in combination with Gibbs artifacts, since the latter are always present (assuming no image filtering).

## Conclusion

It was shown that the contribution made by Gibbs artifacts to DRAs in perfusion studies is very dependent on the position of the subendocardial wall inside the pixel in the absence of zero-filled pre-FFT interpolation. Position variations between patient studies and from frame to frame in a typical ECG-gated perfusion study can explain some of the variability often seen in DRAs. This work also showed that image-based pixel interpolation does not reduce this source of DRA variability. However, interpolation by zero-filling prior to FFT makes the DRA appearance less variable, i.e. reduces the artifact's ringing dependency on the position of the subendocardial wall in relation to image pixels.

## Competing interests

The authors declare that they have no competing interests.

## Authors' contributions

All authors contributed in the design, and intellectual conception. PF, PG, PK, CBD, and DF participated in revision, analysis and interpretation of the data. PF wrote the numerical simulations and shifting algorithm. PF and PG acquired the phantom data and image processed the perfusion raw-data. CBD acquired all the original *in vivo *perfusion data.

## Supplementary Material

Additional file 1 **Animation 1**. Animated GIF showing 4 consecutive time-frames of the basal slice during the Gd bolus arrival in the LV of one patient. The animation alternates between the images that show the shift where the DRAs located in the inferior and anterior segments are more visible, and the images with the shift that shows the highest reduction of the same artifacts. This animation enables an easier visual comparison between the two different shifts. Note also that there is a mild perfusion defect in the inferior-septal segment but both the inferior and anterior segments dark rims are DRAs lasting only for four heart-beats while the real perfusion defect stays visible for much longer. Click here for file
