# Supplementary figures and images for: Variability of myocardial perfusion dark rim Gibbs artifacts due to sub-pixel shifts
Source: J Cardiovasc Magn Reson. 2009 May 27;11(1):17. doi: 10.1186/1532-429X-11-17 (PMC2693509; doi:10.1186/1532-429X-11-17)

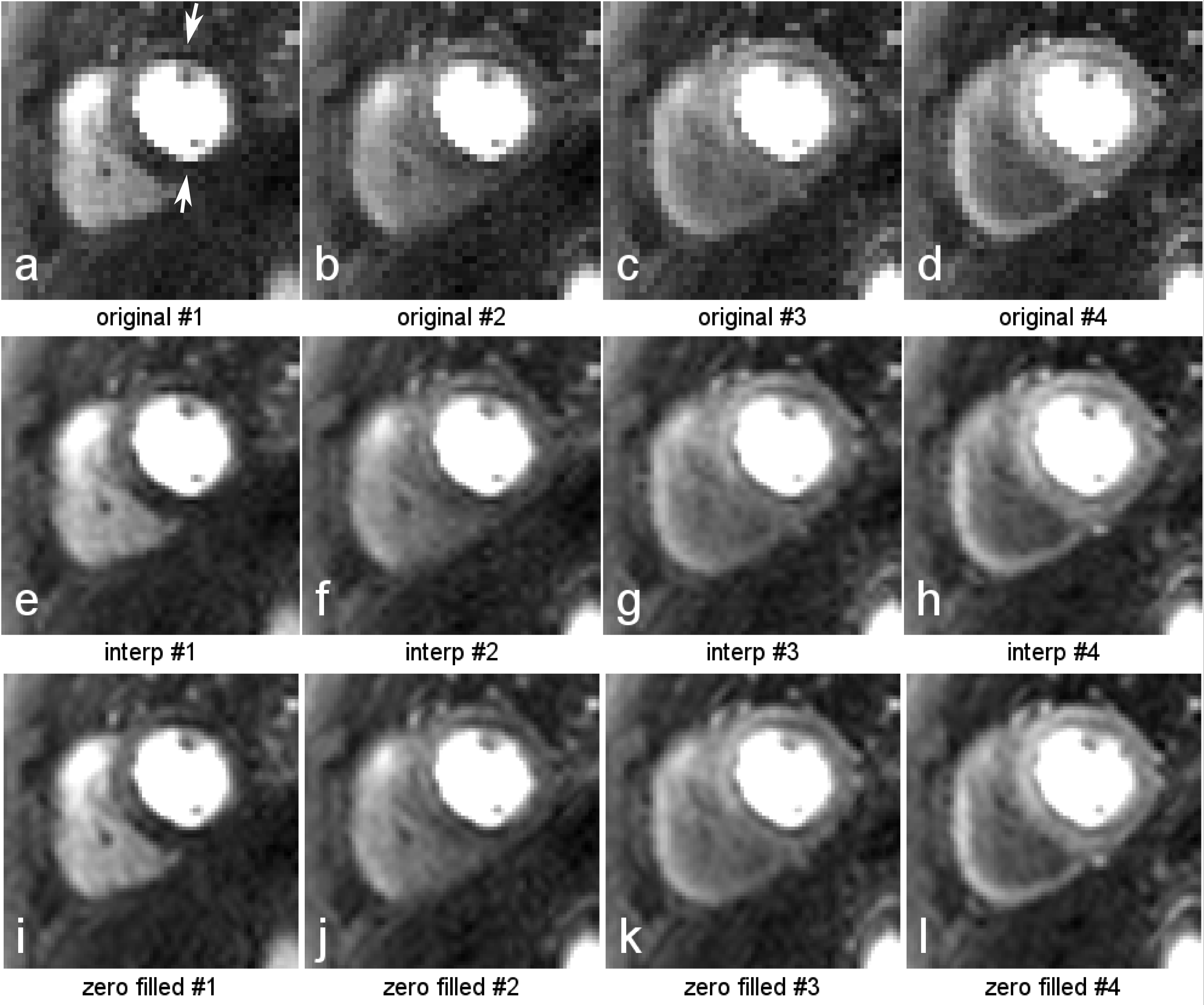

Supplement: Additional file 1 — Animation 1. Animated GIF showing 4 consecutive time-frames of the basal slice during the Gd bolus arrival in the LV of one patient. The animation alternates between the images that show the shift where the DRAs located in the inferior and anterior segments are more visible, and the images with the shift that shows the highest reduction of the same artifacts. This animation enables an easier visual comparison between the two different shifts. Note also that there is a mild perfusion defect in the inferior-septal segment but both the inferior and anterior segments dark rims are DRAs lasting only for four heart-beats while the real perfusion defect stays visible for much longer. [file 1532-429X-11-17-S1.gif]
